# Draft Genome Sequence of a *Pseudomonas* sp. Strain Carrying *bla*_IMP-25_ and *bla*_VIM-2_ Carbapenemase Genes from Hospital Sewage

**DOI:** 10.1128/genomeA.01027-16

**Published:** 2016-10-20

**Authors:** Yiyi Hu, Wenjing Wu, Yu Feng, Xiaoxia Zhang, Zhiyong Zong

**Affiliations:** a Center of Infectious Diseases, West China Hospital, Sichuan University, Chengdu, China; b Division of Infectious Diseases, State Key Laboratory of Biotherapy, Chengdu, China

## Abstract

*Pseudomonas* strain WCHP16 recovered from hospital sewage in West China Hospital, Chengdu, China was found to carry two carbapenemase genes *bla*_IMP-25_ and *bla*_VIM-2_. Here, we report its 5.7-Mb draft genome sequence, comprising 141 contigs and an average 59.53% G+C content. The genome contained 5,504 coding sequences and 67 tRNA genes.

## GENOME ANNOUNCEMENT

The genus *Pseudomonas* comprises 231 species (http://www.bacterio.net/pseudomonas.html)*,* many of which are important opportunistic pathogens for plants and animals. *Pseudomonas* sp. strain WCHP16 was recovered from the influx mainstream of the wastewater treatment plant in West China Hospital, Chengdu, western China, in January 2015. Strain WCHP16 exhibited high-level resistance to meropenem (MIC, >512 µg/ml). Screening for acquired carbapenem-hydrolyzing β-lactamase (carbapenemase) genes *bla*_GES_ (including noncarbapenemase variants), *bla*_IMP_, *bla*_IMI_, *bla*_KPC_, *bla*_NDM_, *bla*_OXA-48_-like, and *bla*_VIM_ was performed using PCR as described previously (1–4). Strain WCHP16 was positive to both *bla*_IMP_ and *bla*_VIM_ and was therefore subjected to whole-genome sequencing.

Genomic DNA of strain WCHP16 was prepared using the QIAamp DNA minikit (Qiagen, Hilden, Germany) and then was sequenced using the Hiseq 2500 Sequencer (Illumina, San Diego, CA, USA) with the 150-bp paired-end protocol and 100× coverage. A total of 4,244,316 reads and 636,647,400 clean bases were generated. The Spades program (version 2.8) (5) was used for *de novo* assembly and generated 141 contigs ≥1,000 bp in length (*N*_50_, 111,079 bp) with a 59.53% G+C content. The genome size was about 5.7 Mb. Annotation of the genomic sequence was carried out using the Prokka program (version 1.11) (6). The genome of strain WCHP16 contained 5,504 coding sequences and 67 tRNA genes. Antimicrobial resistance genes were predicted using ResFinder from the Center for Genomic Epidemiology (http://genomicepidemiology.org/). Strain WCHP16 had two carbapenemase genes, i.e., *bla*_IMP-25_ and *bla*_VIM-2_. *bla*_IMP-25_ was a variant of *bla*_IMP-1_ and was first found in a *Pseudomonas aeruginosa* from South Korea (GenBank accession number EU541448). *bla*_IMP-25_ was rarely reported and had only been identified in *P. aeruginosa* in China and South Korea before (7). Furthermore, to our knowledge, the co-existence of *bla*_IMP-25_ and *bla*_VIM-2_ in a single strain has not been reported before.

Other antimicrobial resistance genes identified in strain WCHP16 included *aacA4* (mediating resistance to aminoglycosides), *arr-2* (to rifampin), *bla*_OXA-1_ (to penicillins), *catB3* (to phenicol), *dfrA22* and *dfrB1* (to trimethoprim), *qnrVC1* (to quinolones), and *sul1* (to sulfonamides).

The 16S rRNA gene sequence of strain WCHP16 had the highest identity with those of *Pseudomonas* sp. 5 (GenBank accession number NZ_JYOC00000000; 99.48% identity)*, Pseudomonas putida* UASWS0946 (NZ_JXOG00000000; 99.35% identity), and *Pseudomonas* sp. NBRC 111117 (NZ_BCAT00000000; 99.35% identity).

Average nucleotide identity (ANI) has been increasingly employed to determine bacterial species and the ≥95% ANI cutoff has been proposed to define *Pseudomonas* species (8, 9) previously. The ≥95% ANI cutoff corresponds to the ≥70% DNA-DNA hybridization value for defining a bacterial species (10). Pair-wise ANI between strain WCHP16 and the three *Pseudomonas* strains was therefore determined using the JSpecies web program (http://imedea.uib-csic.es/jspecies/) with the default settings (11). The genome of strain WCHP16 shared only 79.99% to 81.06% ANI with the three *Pseudomonas* strains, which were far below the ≥95% ANI cutoff. The ANI results suggest that strain WCHP16 is likely to belong to a new species of the *Pseudomonas* genus.

### Accession number(s).

This whole-genome shotgun project has been deposited at DDBJ/EMBL/GenBank under the accession number MBPN00000000. The version described in this paper is the first version, MBPN01000000.
